# Publisher Correction: Pharmacological reactivation of MYC-dependent apoptosis induces susceptibility to anti-PD-1 immunotherapy

**DOI:** 10.1038/s41467-019-08956-x

**Published:** 2019-02-20

**Authors:** Heidi M. Haikala, Johanna M. Anttila, Elsa Marques, Tiina Raatikainen, Mette Ilander, Henna Hakanen, Hanna Ala-Hongisto, Mariel Savelius, Diego Balboa, Bjoern Von Eyss, Vilja Eskelinen, Pauliina Munne, Anni I. Nieminen, Timo Otonkoski, Julia Schüler, Teemu D. Laajala, Tero Aittokallio, Harri Sihto, Johanna Mattson, Päivi Heikkilä, Marjut Leidenius, Heikki Joensuu, Satu Mustjoki, Panu Kovanen, Martin Eilers, Joel D. Leverson, Juha Klefström

**Affiliations:** 1 Cancer Cell Circuitry Laboratory, Research Programs Unit/Translational Cancer Biology and Medicum, University of Helsinki, Street address: Haartmaninkatu 8, P.O. Box 63, 00014 Helsinki, Finland; 2 Hematology Research Unit Helsinki, Department of Clinical Chemistry and Hematology, University of Helsinki and Helsinki University Hospital Comprehensive Cancer Center, Haartmaninkatu 8, 00290 Helsinki, Finland; 3 Research Programs Unit/Molecular Neurology, Biomedicum Stem Cell Center, University of Helsinki, Haartmaninkatu 8, 00290 Helsinki, Finland; 4 Leibniz Institute of Age Research, Fritz Lipmann Institute e.V, Beutenbergstraße 11, 07745 Jena, Germany; 5 Department of Biosciences and Institute of Biotechnology, University of Helsinki, Viikinkaari 5, 00790 Helsinki, Finland; 6 Oncotest GmbH, (Now part of Charles River Laboratories Inc, 251 Ballardvale St, Wilmington, MA 01887, USA), Freiburg, Germany; 7 Institute for Molecular Medicine Finland (FIMM), University of Helsinki, Tukholmankatu 3, 00290 Helsinki, Finland; 8 Department of Mathematics and Statistics, University of Turku, Vesilinnantie 5, 20500 Turku, Finland; 9 Research Programs Unit / Translational Cancer Biology & Medicum, University of Helsinki, P.O. Box 63, (Street address: Haartmaninkatu 8), 00290 Helsinki, Finland; 10 Department of Oncology, University of Helsinki and Helsinki University Hospital, Haartmaninkatu 4, 00290 Helsinki, Finland; 11 Department of Pathology, University of Helsinki and Helsinki University Hospital, Haartmaninkatu 3, 00290 Helsinki, Finland; 12 Breast Surgery Unit, Helsinki University Hospital, Kasarmikatu 11-13, 00290 Helsinki, Finland; 13 Department of Pathology, HUSLAB and Haartman Institute, University of Helsinki and Helsinki University Hospital, Haartmaninkatu 3, 00290 Helsinki, Finland; 14 Theodor Boveri Institute and Comprehensive Cancer Center Mainfranken, Biocenter, University of Würzburg, Am Hubland, D-970074 Germany; 15 Oncology Development, AbbVie, Inc., 1 N Waukegan Road, North Chicago, 60064 IL USA; 16 Present Address: Department of Medical Oncology, Dana-Farber Cancer Institute and Harvard Medical School, 360 Longwood Ave, 02215 Boston, MA USA

Correction to: *Nature Communications*; 10.1038/s41467-019-08541-2, published online 06 February 2019

The original version of this Article contained an error in Fig. 7. In panel b, the survival curves were shifted relative to the *y* axis. This error has been corrected in both the PDF and HTML versions of the Article.

