# Supplementary material for: Pharmacological reactivation of MYC-dependent apoptosis induces susceptibility to anti-PD-1 immunotherapy
Source: Nat Commun. 2019 Feb 6;10:620. doi: 10.1038/s41467-019-08541-2 (PMC6365524; doi:10.1038/s41467-019-08541-2)
Supplement: Supplementary file 1 — Supplementary Information [file 41467_2019_8541_MOESM1_ESM.pdf]

# Supplementary Information

**Haikala et al.,**

## Pharmacological reactivation of MYC-dependent apoptosis induces susceptibility to anti-PD-1 immunotherapy

## SUPPLEMENTARY FIGURES

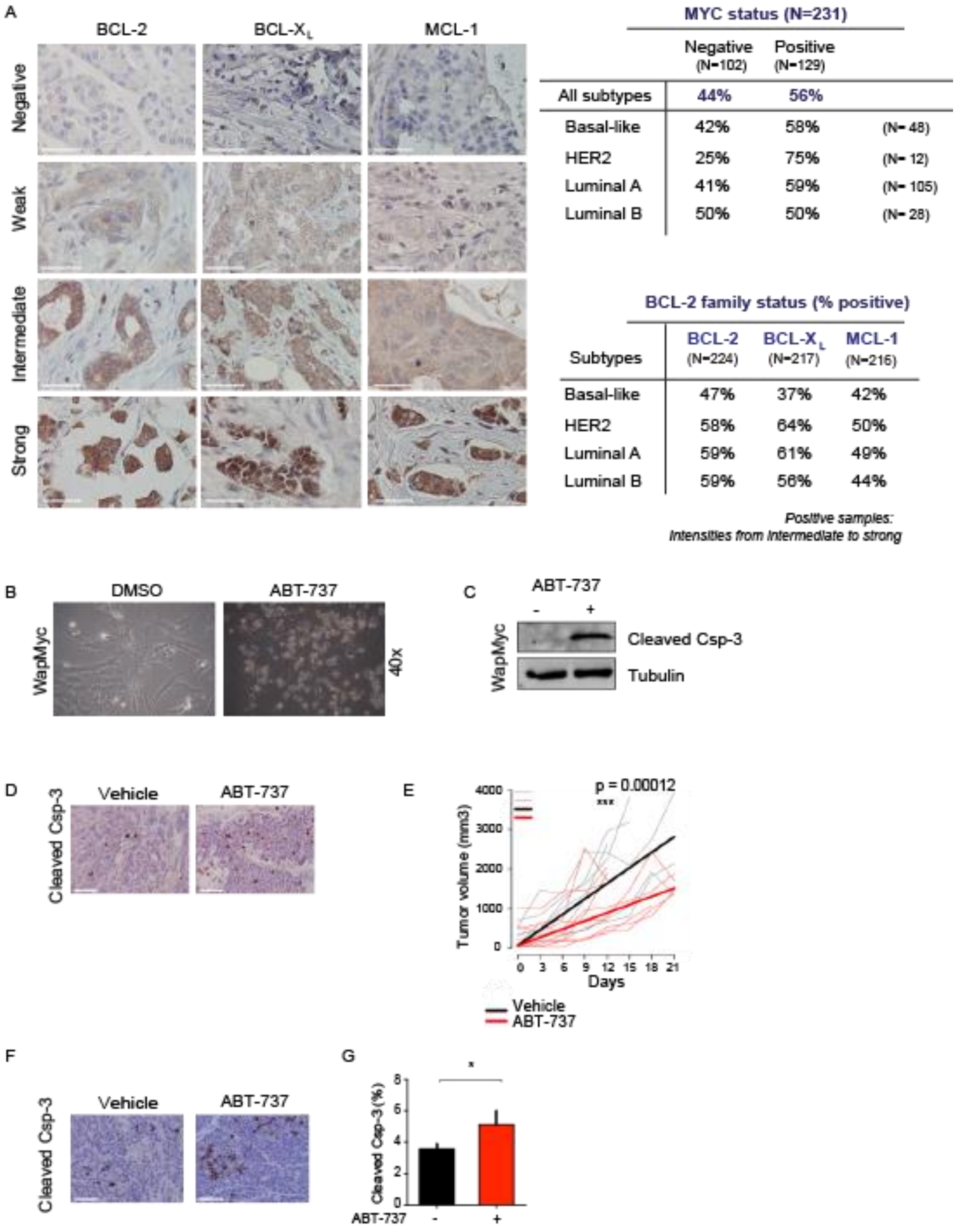


**Supplementary Figure 1. BCL-2 family proteins in breast cancer subtypes and the efficacy of ABT-737 in breast cancer models**

(A) Representative images of immunostainings (left) and the BCL-2/BCL-$X_L$/MCL-1 status in breast cancer samples (right). (B) ABT-737-induced apoptosis in isolated WapMyc tumor cells. Cells were treated with vehicle (DMSO) or 1 µM ABT-737 for 24 hours. (C) Cleaved caspase-3 in primary cultures of WapMyc tumor cells treated for 24 hr with DMSO or 1 µM ABT-

737. (D) ABT-737-induced apoptosis in WapMyc tumors. (E) Statistical modeling of the treatment effect. The mixed-effects modeling framework shows longitudinal tumor growth profiles for vehicle and ABT-737 treated mice. Fisher's exact test. (F) Apoptosis in ABT-737 treated syngrafted tumors. (G) Quantification of apoptosis. Student's *t*-test (unpaired), SD.

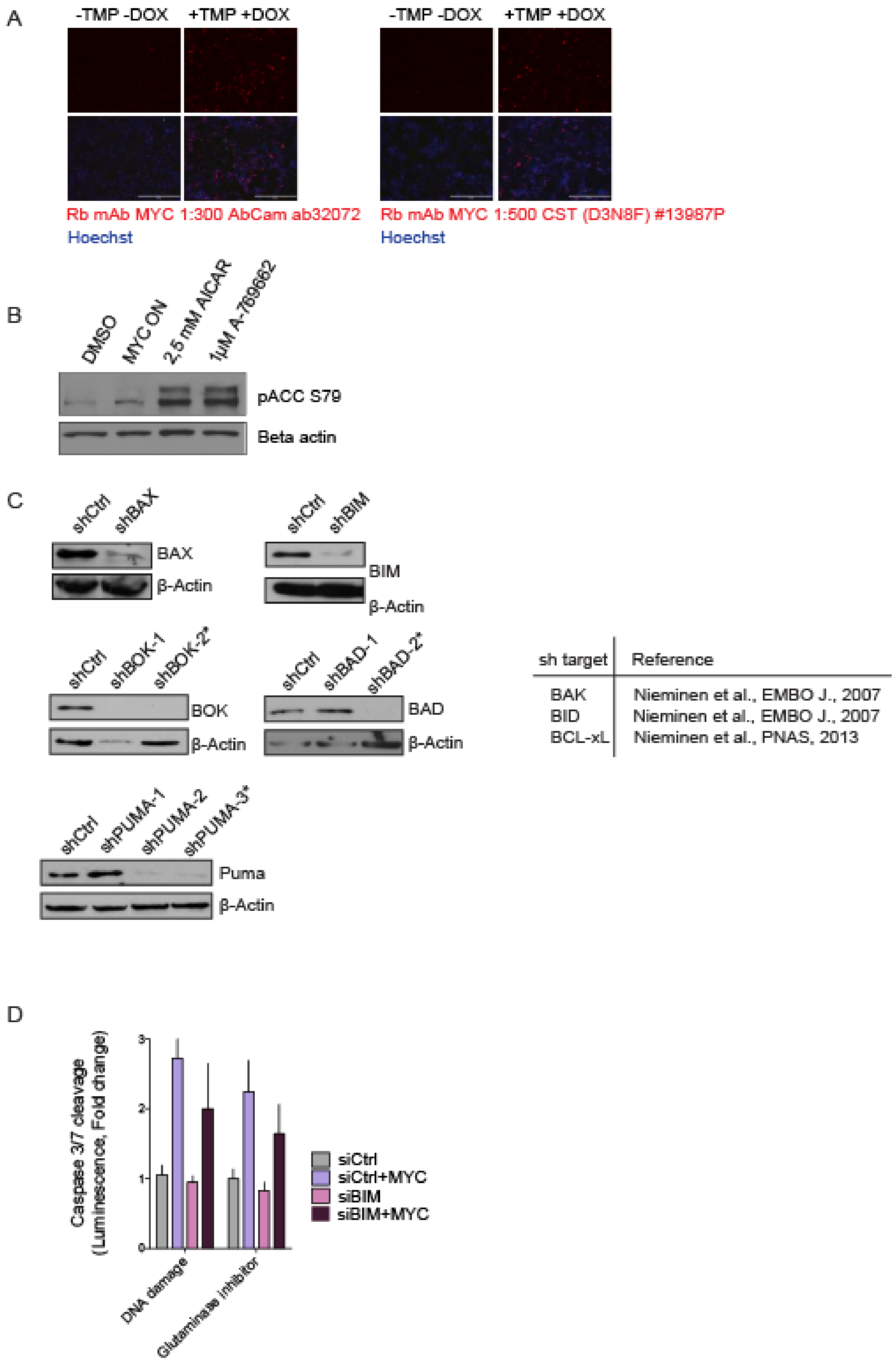


**Supplementary Figure 2. CRISPR activation of MYC, validation of shRNAs and role of BIM in MYC-dependent apoptosis**

(A) Immunofluorescence staining of MYC in HEK-293 CRISPR-act MYC cells with or without MYC activation. Similar results were obtained with two different

antibodies (indicated in the figure). (B) A-769662 activates AMPK in MCF10A MycER cells. Cells were treated with vehicle (DMSO) or 1 μM A-769662 for 24 hr to activate AMPK. MYC activation (100 nM 4OHT, 24 hr) and 2.5 mM AICAR 24 hr were used as positive controls for AMPK activation. pACC S79: Marker for AMPK activity. Beta actin: Loading control. (C) Knockdown validation of BCL-2-family targeted shRNAs. shRNAs marked with a star were chosen for the screen (**Fig.3B**) Beta actin serves as loading control. shRNAs indicated in the table (right side) have been previously published. (D) Silencing of BIM protects cells from MYC-dependent apoptosis. Cells were treated with 0.1 μg/ml doxorubicine or 1 μM BPTES (glutaminase inhibitor) for 24 hr before apoptosis detection. Error bars show SD.

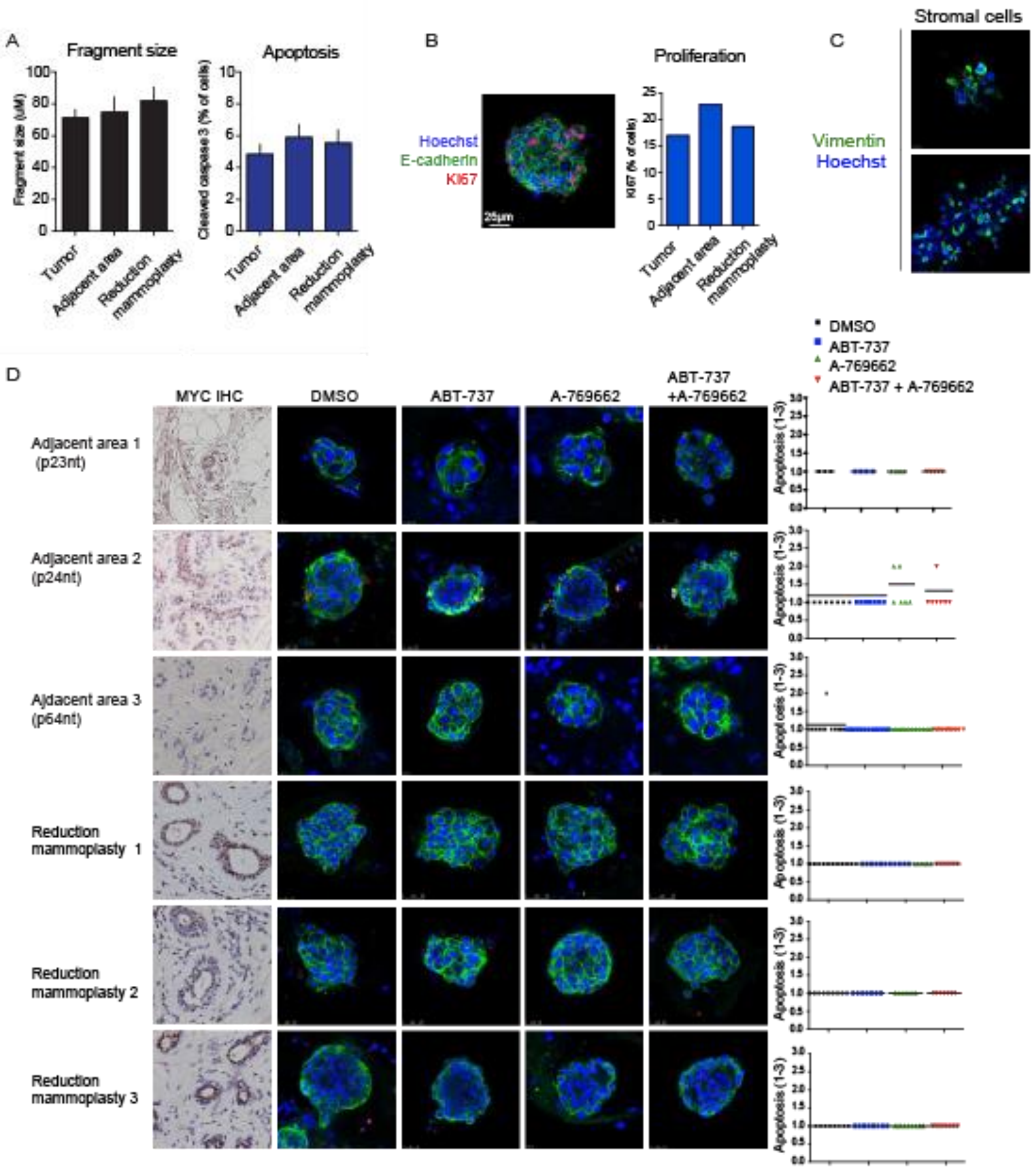


**Supplementary Figure 3. Characterization of patient-derived explant cultures and effects of AB treatment in non-tumorous tissues**

(A) Patient-derived explant cultures (PDEc) from different origins (tumor, adjacent area or reduction mammoplasty) are similar in size and show negligible level of basal apoptosis. Fragment size was determined from scale bars of immunofluorescence images. N = 13 tumors (97 explants), 9 adjacent areas (55 explants), 6 reduction mammoplasty samples (50 explants). The percentage of apoptotic cells was determined from cleaved caspase-3 stainings. N = 12 tumors (64 explants), 7 adjacent areas (39 explants), 6 reduction mammoplasty samples (56 explants). Error bars indicate SD. (B) Proliferation in PDEc counted from Ki67 stainings. (C) PDEc is a heterotypic

culture. Immunofluorescence stainings of vimentin-positive stromal cells in the culture. (D) AB treatment does not induce apoptosis in PDEcs representing healthy mammary epithelial cells. 3 adjacent areas and 3 reduction mammoplasty samples were adjusted to culture for 6 days and treated for 24 hr with vehicle (DMSO), 1μM ABT-737, 10 μM A-769662 or ABT-737+A-769662 combination, and subsequently immunostained for cleaved caspase-3. The level of apoptosis was scored from confocal immunofluorescence microscopy images. The panel (left) shows representative images. The graphs (right) show quantification of apoptosis in the fragments. Apoptosis was scored as 1 = <10% apoptotic cells/explant, 2 = >10% apoptotic cells/explant in cohesive structure, 3 = >10% apoptotic cells/explant in deteriorated dying structure. In the graphs each dot indicates one fragment. The horizontal lines indicate average.

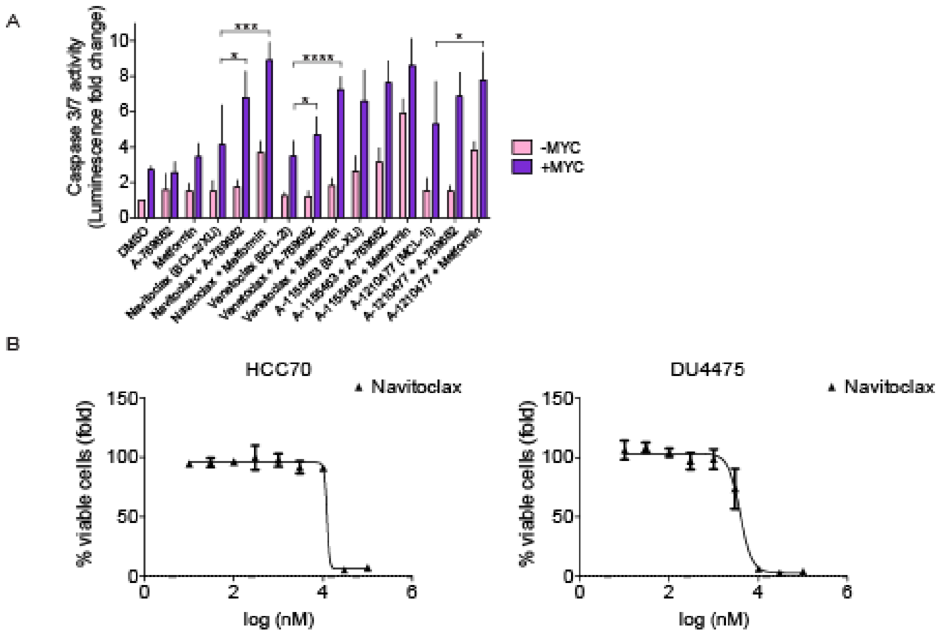


C

| Cell line | p53 mutation status | MYC protein level | Growth inhibition Navitoclax EC50 (uM) | Navitoclax EC20 + 3 mM Metformin | Navitoclax EC20 + 10 mM Metformin | Navitoclax EC20 + 30 uM A-769662 | Navitoclax EC20 + 100 uM A-769662 |
|---|---|---|---|---|---|---|---|
| HCC70 | R248Q | 0 | 12.154 | - ns/ns | - *** /ns | - ns/ns | - ns/ns |
| HCC1143 | R248Q | 0 | 3.671 | - ns/ns | N/A | - ns/ns | - ns/ns |
| HCC38 | R273L | 0 | 1.171 | - ***/ns | - *** /ns | - *** /ns | + *** /* |
| MDA-MB-453 | WT | 0 | 4.134 | + **** /**** | + **** /**** | + **** /*** | + **** /**** |
| HCC1395 | R175H | 0 | 23.774 | - ns/ns | - ns/ns | - ns/ns | - ns/ns |
| MDA-MB-468 | R273H | + | 1.138 | + **** /**** | - ns/**** | - * /ns | + **** /**** |
| Hs578T | V157F | + | 3.877 | + ** /** | + **** /*** | - ** /ns | - ns/ns |
| MDA-MB-157 | del / frameshift | + | 2.554 | + ** /*** | + **** /**** | - ns/ns | + * /**** |
| HCC1187 | del | + | 8.100 | + ** /**** | + **** /**** | + ** /*** | + * /**** |
| BT-20 | K132Q | + | 5.023 | + ** /*** | - ** /ns | + ** /**** | + * /**** |
| HCC1937 | R306* / nonsense | + | 8.007 | - ns/ns | + **** /* | - ns/ns | + ** /* |
| MDA-MB-231 | R280K | ++ | 0.231 | + *** /**** | - ns/**** | + **** /**** | + **** /**** |
| DU4475 | WT | ++ | 3.868 | + **** /**** | + **** /**** | + ** /* | + **** /**** |
| MDA-MB-436 | ins / controversial | +++ | 3.207 | + * /** | - ns/**** | + *** /* | + *** /** |
| BT-549 | R249S | +++ | 7.191 | - ns/ns | + *** /*** | + * /** | - ns/** |
| HCC1806 a) | ins | +++ | 12.727 | - ns/ns | + **** /*** | - ns/ns | - ns/ns |
| MCF10A MycER +4OHT | WT | +++ | 1.148 | + **** /**** | + **** /**** | + *** /** | + **** /**** |

Reference:
IARC Tp53 database
COSMIC Cell Line database

| | |
|---|---|
| + | Combination reduces cell viability more than Navitoclax EC20 or Metformin or A-769662 |
| - | No significant synergy detected with combination treatments compared to Navitoclax EC20 or Metformin or A-769662 |
| - | Metformin or A-769662 alone reduces cell viability more than 80 % |

a) acantholytic squamous carcinoma

Statistical significance: Metformin or A-769662 is compared to the combination treatment / Navitoclax EC20 alone is compared to the combination treatment
Statistical significance tested with unpaired Student's t-test, where P-values are presented as follows:
ns $P > 0.05$, * $P \leq 0.05$, ** $P \leq 0.01$, *** $P \leq 0.001$, **** $P \leq 0.0001$

MYC protein level

| | |
|---|---|
| 0 | 0-0.1 |
| + | 0.1-0.4 |
| ++ | 0.41-0.8 |
| +++ | 0.8-1.0 |

**Supplementary Figure 4. Efficacy of BH3 mimetic molecules in MCF10A cells and AB treatment effects in TNBC cell lines**

(A) AMPK activation together with different BH3 mimetics induces MYC-dependent apoptosis. MYC was activated in MCF10A MycER cells for 24 hr with 100 nM 4OHT followed by 24 hr treatment with 100 nM navitoclax (BCL-2/$X_L$ inhibitor), venetoclax (BCL-2 inhibitor), A-1155463 (BCL-$X_L$ inhibitor), and A-1210477 (MCL-1 inhibitor), or in combinations with either 1 μM A-

769662 or 10 mM metformin. Student's *t*-test (unpaired), SD. (B) Example kill curves. HCC70 and DU4475 were treated with increased concentrations of navitoclax to determine $EC_{20}$. (C) Primary data from experiments performed to determine effect of combination treatments in 16 TNBC cell lines and the MCF10A MycER control cell line. The figure shows p53 mutation status, relative MYC protein levels (quantified from western blot analysis), navitoclax $EC_{50}$, and combination effect with AMPK activating compounds at navitoclax $EC_{20}$.

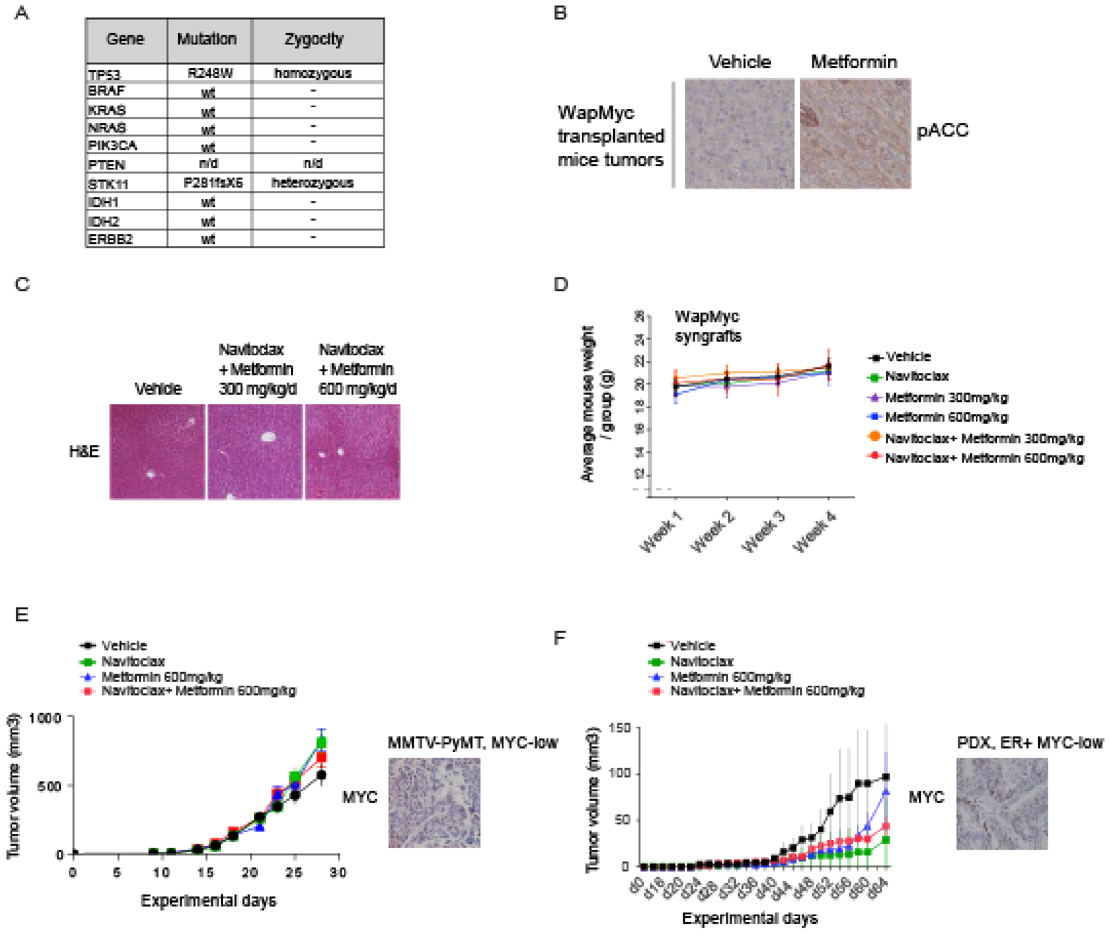

| Gene | Mutation | Zygocity |
|---|---|---|
| TP53 | R248W | homozygous |
| BRAF | wt | - |
| KRAS | wt | - |
| NRAS | wt | - |
| PIK3CA | wt | - |
| PTEN | n/d | n/d |
| STK11 | P281fsX6 | heterozygous |
| IDH1 | wt | - |
| IDH2 | wt | - |
| ERBB2 | wt | - |



**Supplementary Figure 5. Supplementary *in vivo* data**

(A) Mutation profile of TNBC-PDX tumor. (B) Metformin activates AMPK *in vivo*. IHC-stained tumor sections from WapMyc syngrafts treated with either vehicle or 300 mg/kg meformin. pACC is an AMPK activity marker. (C) AB treatment does not affect the liver morphology in treated mice. Representative images from H&E staining of livers. (D) AB treatment does not affect the weight of the mice. Mice were weighed in the beginning of each experimental week to monitor their well-being, and no differences between the different treatment groups were found. (E) Efficacy of navitoclax + metformin treatment in MMTV-PyMT syngraft model with low MYC expression status. Immunohistochemical staining of MYC shown in the right. (F) Efficacy of navitoclax + metformin treatment in MYC-low ER+ PDX model. Immunohistochemical staining of MYC shown in the right.

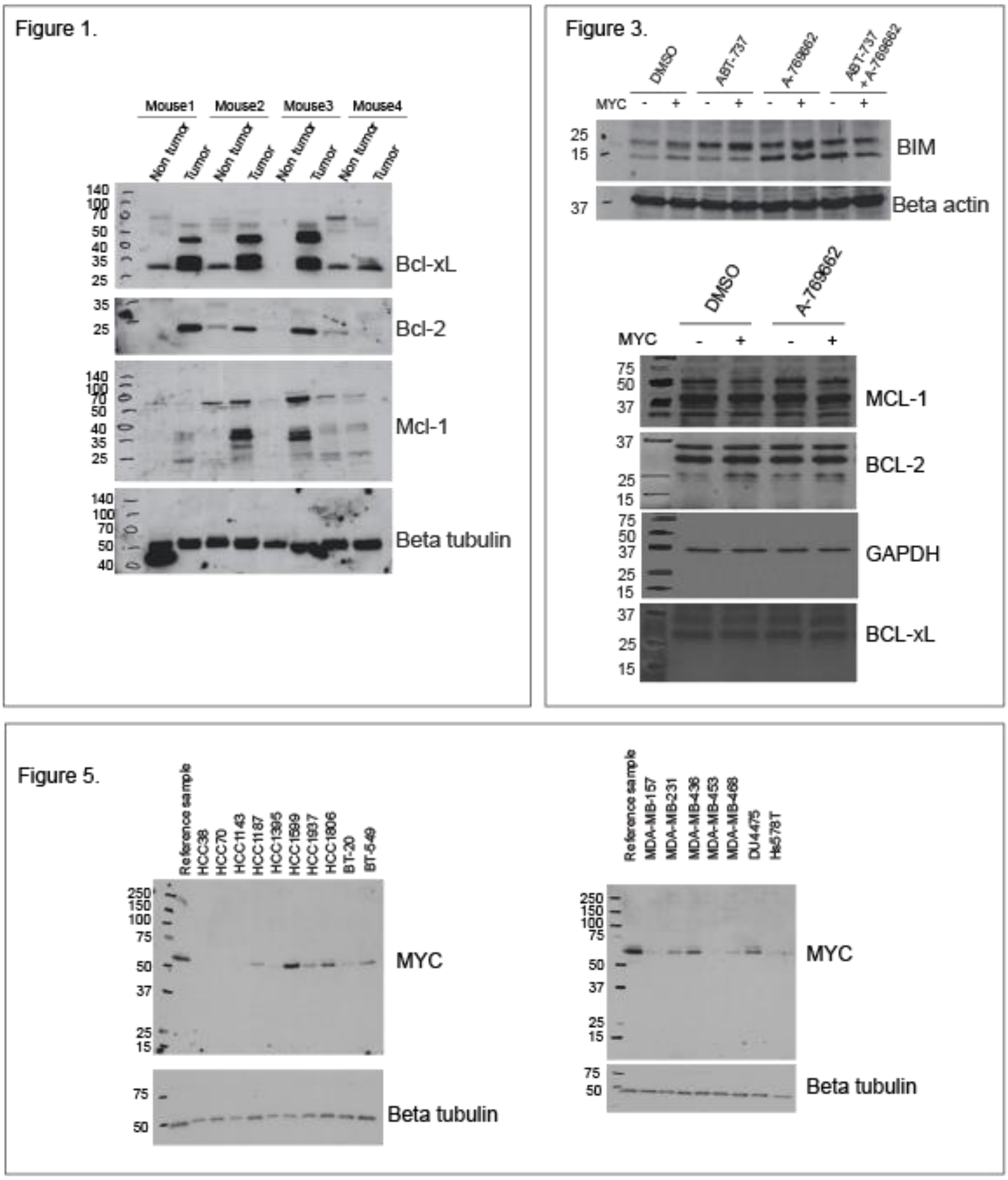


**Supplementary Figure 6.** Western blots with original ladders.

| Tumor code | Diagnosis | Tumor grade (G1-G3) | MIB | Receptor status | MYC status (IHC) | p53 status | ABT-737 + A-769662 response (1-3) |
|---|---|---|---|---|---|---|---|
| T1 | Carcinoma ductale | G3 | 3 | Her2+ | High | Missense mutation | 3.0 |
| T2 | Carcinoma tubulare | G1 | 1 | ER+ | High | WT | 3.0 |
| T3 | Carcinoma apocrinum | G3 | 3 | Her2+ | High | Intronic mutation | 2.3 |
| T4 | Carcinoma ductale | G3 | 3 | TNBC | High | WT | 2.7 |
| T5 | Carcinoma ductale | G3 | 3 | ER+,PR+,Her2+ | High | WT | 2.5 |
| T6 | Carcinoma ductale et lobulare | G2 | 2 | ER+,Her2+ | Low | WT | 1.3 |
| T7 | Carcinoma ductale | G3 | 3 | ER+,PR+,Her2+ | Low | WT | 1.4 |
| T8 | Carcinoma lobulare | G3 | 2 | ER+,PR+,Her2+ | Low | WT | 1.8 |
| T9 | Carcinoma ductale | G1 | 1 | ER+,PR+,Her2+ | Low | WT | 2.3 |
| T10 | Carcinoma ductale | G1 | 3 | TNBC | Low | WT | 2.0 |

| Tumor code | Diagnosis | Tumor grade (G1-G3) | MIB | Receptor status | MYC status (IHC) | p53 status | Navitoclax + Metformin response (1-3) |
|---|---|---|---|---|---|---|---|
| T11 | Carcinoma lobulare | G3 | 3 | ER+, Her2+ | High | N/D | 2.5 |
| T12 | Carcinoma ductale | G3 | 2 | ER+, PR+, Her2+ | High | N/D | 2.8 |
| T13 | Carcinoma lobulare | G3 | 3 | ER+, PR+, Her2+ | High | N/D | 2.4 |
| T14 | Carcinoma ductale | G2 | 3 | ER+, PR+ | High | N/D | 3.0 |
| T15 | Carcinoma ductale | G3 | 3 | ER+, PR+, Her2+ | High | N/D | 3.0 |

**Supplementary Table 1.** Diagnosis, MYC status, P53 status, and AB response in PDEc samples.

| Cell line name | ATCC Cat. No. | Culture medium components |
|---|---|---|
| HCC38 | CRL-2314 | RPMI-1640 (ATCC 30-2001) + 10% fetal bovine serum (Biowest) |
| HCC70 | CRL-2315 | RPMI-1640 (ATCC 30-2001) + 10% fetal bovine serum (Biowest) |
| HCC1143 | CRL-2321 | RPMI-1640 (ATCC 30-2001) + 10% fetal bovine serum (Biowest) |
| HCC1187 | CRL-2322 | RPMI-1640 (ATCC 30-2001) + 10% fetal bovine serum (Biowest) |
| HCC1395 | CRL-2324 | RPMI-1640 (ATCC 30-2001) + 10% fetal bovine serum (Biowest) |
| HCC1599 | CRL-2331 | RPMI-1640 (ATCC 30-2001) + 10% fetal bovine serum (Biowest) |
| HCC1806 | CRL-2335 | RPMI-1640 (ATCC 30-2001) + 10% fetal bovine serum (Biowest) |
| HCC1937 | CRL-2336 | RPMI-1640 (ATCC 30-2001) + 10% fetal bovine serum (Biowest) |
| BT-20 | HTB-19 | EMEM (ATCC 30-2003) + 10% fetal bovine serum (Biowest) |
| BT-549 | HTB-122 | RPMI-1640 (ATCC 30-2001) + 10% fetal bovine serum (Biowest) + 0.023 IU/ml insulin (Sigma) |
| Hs578T | HTB-126 | DMEM (ATCC 30-2002) + 10% fetal bovine insulin (Biowest) + 0.01 mg/ml insulin (Sigma) |
| DU4475 | HTB-123 | RPMI-1640 (ATCC 30-2001) + 10% fetal bovine serum (Biowest) |
| MDA-MB-157 | HTB-24 | Leibovitz’s L-15 (ATCC 30-2008) + 10% fetal bovine serum (Biowest) |
| MDA-MB-231 | HTB-26 | Leibovitz’s L-15 (ATCC 30-2008) + 10% fetal bovine serum (Biowest) |
| MDA-MB-436 | HTB-130 | Leibovitz’s L-15 (ATCC 30-2008) + 10% fetal bovine serum (Biowest) |
| MDA-MB-453 | HTB-131 | Leibovitz’s L-15 (ATCC 30-2008) + 10% fetal bovine serum (Biowest) + 10 µg /ml insulin (Sigma) + 16 µg/ml glutathione (Sigma) |
| MDA-MB-468 | HTB-132 | Leibovitz’s L-15 (ATCC 30-2008) + 10% fetal bovine serum (Biowest) |

**Supplementary Table 2.**

Culture conditions for the 17 TNBC cell lines (ATCC panel)

| | Western Blot | IHC | Immunofluorescence |
|---|---|---|---|
| **MYC** | 9E10, Biolegend | Y69, ab32072, Abcam | Y69, ab32072, Abcam |
| **BCL-2** | #2876, Cell Signaling Technology | N-19, sc-492, Santa Cruz Biotechnology | - |
| **BCL-$X_L$** | 551022, BD Biosciences | #2764, Cell Signaling Technology | - |
| **CD4** | - | ab183685, Abcam | - |
| **CD8** | - | bs-0648R, BIOSS | - |
| **MCL-1** | 600-401-394, Rockland | S-19, Santa Cruz Biotechnology | - |
| **Cleaved Caspase-3** | Asp175, #9661, Cell Signaling Technology | ab13847, Abcam | Asp175, #9661, Cell Signaling Technology |
| **BIM** | #2819, Cell Signaling Technology | C34C5, #2933, Cell Signaling Technology | C34C5, #2933, Cell Signaling Technology |
| **Vinculin** | V9131, Sigma | - | - |
| **ACC** | #3676, Cell Signaling Technology | - | - |
| **pACC S79** | #3661, Cell Signaling Technology | D7D11, #11818, Cell Signaling Technology | - |
| **E-cadherin** | - | - | 36/E-Cadherin, BD Biosciences |
| **Lamin B** | C-20, sc-6216, Santa Cruz Biotechnology | - | - |
| **BOK** | #4521, Cell Signaling Technology | - | - |
| **BAD** | #9292, Cell Signaling Technology | - | - |
| **PUMA** | #4976, Cell Signaling Technology | - | - |
| **GAPDH** | 14C10, #2118, Cell Signaling Technology | - | - |
| **Beta Tubulin** | T7451, Sigma Aldrich | - | - |
| **Vimentin** | - | - | Vim 3B4, ab28028, AbCam |

**Supplementary table 3.** Antibody list.

| Gene | Sequence 5’-3’ | Species | Assay |
|---|---|---|---|
| **sgMyc1** | CCCTTTATAATGCGAGGGTC | Human | CRISPR activation |
| **sgMyc2** | TCTCGCTAATCTCCGCCCAC | Human | CRISPR activation |
| **sgMyc3** | GGTTCCCAAAGCAGAGGGCG | Human | CRISPR activation |
| **sgMyc4** | AGCTAGAGTGCTCGGCTGCC | Human | CRISPR activation |
| **sgMyc5** | GCGCGCGTAGTTAATTCATG | Human | CRISPR activation |
| **TP53** | 1F: TCTCGGCTCCGTGTATTTTC | Human | Long-range sequencing |
| **TP53** | 1R: TACAAATGTGCCAGGCTGAA | Human | Long-range sequencing |
| **TP53** | 2F: TTGGTTCTGGGACTCCTCAC | Human | Long-range sequencing |
| **TP53** | 2R: CTCCGTCATGTGCTGTGACT | Human | Long-range sequencing |
| **TP53** | 3F: GTTTCTTTGCTGCCGTCTTC | Human | Long-range sequencing |
| **TP53** | 3R: GTGGTTTCAAGGCCAGATGT | Human | Long-range sequencing |

**Supplementary table 4.** sgRNA and primer sequences used in the study.
