# Supplementary material for: Pharmacological reactivation of MYC-dependent apoptosis induces susceptibility to anti-PD-1 immunotherapy
Source: Nat Commun. 2019 Feb 6;10:620. doi: 10.1038/s41467-019-08541-2 (PMC6365524; doi:10.1038/s41467-019-08541-2)
Supplement: Supplementary file 3 — Reporting Summary [file 41467_2019_8541_MOESM3_ESM.pdf]

nature research

Corresponding author(s): *Double-blind peer review submissions: write DBPR and your manuscript number here instead of author names.*

Last updated by author(s): *YYYY-MM-DD*

Reporting Summary

Nature Research wishes to improve the reproducibility of the work that we publish. This form provides structure for consistency and transparency in reporting. For further information on Nature Research policies, see Authors & Referees and the Editorial Policy Checklist.

Statistics

For all statistical analyses, confirm that the following items are present in the figure legend, table legend, main text, or Methods section.

n/a	Confirmed	
☐	☒	The exact sample size (*n*) for each experimental group/condition, given as a discrete number and unit of measurement
☐	☒	A statement on whether measurements were taken from distinct samples or whether the same sample was measured repeatedly
☐	☒	The statistical test(s) used AND whether they are one- or two-sided<br>*Only common tests should be described solely by name; describe more complex techniques in the Methods section.*
☐	☒	A description of all covariates tested
☐	☒	A description of any assumptions or corrections, such as tests of normality and adjustment for multiple comparisons
☐	☒	A full description of the statistical parameters including central tendency (e.g. means) or other basic estimates (e.g. regression coefficient) AND variation (e.g. standard deviation) or associated estimates of uncertainty (e.g. confidence intervals)
☐	☒	For null hypothesis testing, the test statistic (e.g. *F*, *t*, *r*) with confidence intervals, effect sizes, degrees of freedom and *P* value noted<br>*Give P values as exact values whenever suitable.*
☐	☒	For Bayesian analysis, information on the choice of priors and Markov chain Monte Carlo settings
☐	☒	For hierarchical and complex designs, identification of the appropriate level for tests and full reporting of outcomes
☐	☒	Estimates of effect sizes (e.g. Cohen's *d*, Pearson's *r*), indicating how they were calculated

Our web collection on statistics for biologists contains articles on many of the points above.

Software and code

Policy information about availability of computer code

Data collection	*Provide a description of all commercial, open source and custom code used to collect the data in this study, specifying the version used OR state that no software was used.*
Data analysis	Data was analysed using GraphPad PRISM 5.

For manuscripts utilizing custom algorithms or software that are central to the research but not yet described in published literature, software must be made available to editors/reviewers. We strongly encourage code deposition in a community repository (e.g. GitHub). See the Nature Research guidelines for submitting code & software for further information.

Data

Policy information about availability of data

All manuscripts must include a data availability statement. This statement should provide the following information, where applicable:
- Accession codes, unique identifiers, or web links for publicly available datasets
- A list of figures that have associated raw data
- A description of any restrictions on data availability

Relevant manuscript data is available from the authors upon request.

Field-specific reporting

Please select the one below that is the best fit for your research. If you are not sure, read the appropriate sections before making your selection.

☒ Life sciences ☐ Behavioural & social sciences ☐ Ecological, evolutionary & environmental sciences

October 2018

Life sciences study design

All studies must disclose on these points even when the disclosure is negative.

Sample size	For cell line experiments, at least 3 biological experiments were performed according to the good scientific practice. To optimize the number of mice used in drug treatment experiments, sample size in animal studies was calculated by using The Power Analysis method. Power analysis allows to estimate the smallest number of mice needed to obtain significance.
Data exclusions	In tissue microarray (TMA) analysis amples that were missing from the slide or had insufficient clinical diagnosis data, or had inadequate technical quality were excluded from all analyses. In immunoprofiling experiments, populations under 90 parental cells were excluded from the analysis. Otherwise no exclusions were made.
Replication	Cell line experiments: At least 3 biological replicates were performed per experiment. Animal experiments: WapMyc syngrafting experiment with drug treatments was replicated several times and the results were consistent.
Randomization	Mice were randomized into groups by pooling all animals and then dividing randomly into the cages before the start of the experiment.
Blinding	Imaging samples / slides were blinded for analysis. Tumor measurements were done by at least 2 individual researchers and the result was averaged from the 2 measurements.

Reporting for specific materials, systems and methods

We require information from authors about some types of materials, experimental systems and methods used in many studies. Here, indicate whether each material, system or method listed is relevant to your study. If you are not sure if a list item applies to your research, read the appropriate section before selecting a response.

Materials & experimental systems

n/a	Involved in the study	
☐	☒	Antibodies
☐	☒	Eukaryotic cell lines
☒	☐	Palaeontology
☐	☒	Animals and other organisms
☒	☐	Human research participants
☒	☐	Clinical data

Methods

n/a	Involved in the study	
☒	☐	ChIP-seq
☒	☐	Flow cytometry
☒	☐	MRI-based neuroimaging

Antibodies

Antibodies used	All used antibodies have been described (including catalogue number) in the methods and supplementary methods section.
Validation	Antibodies used for immunohistochemistry were validated in the Helsinki University Hospital pathology core by using negative and positive controls, and serial dilutions of the antibodies. Western blot antibodies were typically validated using positive and negative controls.

Eukaryotic cell lines

Policy information about cell lines

Cell line source(s)	All used cell lines have been described in the methods and supplementary methods section of the manuscript. All cell lines were obtained from ATCC, except HEK293, which were a gift from Prof. Timo Otonkoski (University of Helsinki).
Authentication	Typically low passage cell lines (directly from ATCC) were used to avoid cross-contamination or phenotypic change upon culture. MCF10A MycER cells were validated by western blot and by testing their apoptotic sensitivity (with -glutamine and TRAIL). WapMyc mice were validated by sequencing.
Mycoplasma contamination	We test our cell lines for mycoplasma, and all have been negative.
Commonly misidentified lines (See ICLAC register)	MDA-MB-435 cells were a part of the commercially available ATCC panel of 17 triple-negative breast cancer cell lines. The first passage of cells was immediately expanded and frozen, and these low passage cell lines were used for cell viability assays.

Animals and other organisms

Policy information about studies involving animals; ARRIVE guidelines recommended for reporting animal research

Laboratory animals	Female FVB / FVB WapMyc and female NSG mice were used in the study (described in the methods section).
Wild animals	No wild animals were used in the study.
Field-collected samples	No field samples were used in the study,
Ethics oversight	All animal experiments were approved by the National Animal Ethics Committee of Finland (Licence number: ESAVI-3216/04.10.07/2013), and the mouse colonies were maintained according to the protocols of the Experimental Animal Committee of the University of Helsinki. Ethical and technical quality of the animal experiments were also reviewed and accepted by Abbvie IncPatient tumor samples were collected from consenting patients with permission, approved by the Hospital District of Helsinki and Uusimaa (Ethical permit: 243/13/03/02/2013). .

Note that full information on the approval of the study protocol must also be provided in the manuscript.
